# Correlation between the number and pattern of lateral pterygoid muscle attachments and pathologic changes of the temporomandibular joint according to Hegab stages based on MRI findings of 510 joints

**DOI:** 10.1038/s41598-025-20061-2

**Published:** 2025-09-29

**Authors:** Ayman F. Hegab, Mohammad Shuman, Hossam Abd Al Hameed, Khaled Karam, Mustafa G. Khallaf, Hussien F. Elrawy, Mohammed Elsaid

**Affiliations:** 1 Department of Oral & Maxillofacial Surgery, Faculty of Dental Medicine, Al- Azhar University, Cairo, Egypt; 2 Diagnostic Radiology, Faculty of Medicine for Men, Al Azher University, Cairo, Egypt; 3 OMFS Faculty of Dentistry, Al-Azhar University, Cairo, Egypt; 4 Cleveland Dental Institute, Columbus, OH USA; 5 OMFS, Cairo, Egypt; 6 GPR-Miami Valley Hospital, Dayton, OH USA; 7 Orofacial Pain Program, University of Rochester, Rochester, NY USA; 8 Department of Oral and Maxillofacial Surgery, Faculty of Dentistry, Al-Azhar University, Assuit, Egypt

**Keywords:** TMJ, Internal derangement, Lateral pterygoid muscle, MRI, Musculoskeletal system, Oral anatomy

## Abstract

The correlation between the lateral pterygoid muscle attachment type to the disc-condyle complex and temporomandibular joint (TMJ) dysfunction has rarely been discussed and remains unclear. The study aimed to assess the correlation between the number and pattern of LPM attachment and the pathologic findings of the temporomandibular joint based on MR imaging findings. The study population comprised consecutive TMD patients. They were included if they had TMD requiring MRI examination for evaluation of internal derangement. Patients with either TMJ clicking, TMJ locking, restricted movement of the jaw, or pain in the TMJ region were included in the study. Patients with rheumatoid arthritis, condylar hyperplasia, and congenital craniofacial syndrome, and those who had undergone previous TMJ surgery were excluded from this study. Variations of the number of heads and the attachment pattern of the LPM to DCC was evaluated using MRI in the oblique sagittal and coronal images. The variation of the LPM heads and attachment patterns was correlated with pathologic changes of the TMJ. The sample size calculation was performed using G*Power version 3.1.9.2. The significance level was set at 0.05. The data were analysed using Instat statistical software (GraphPad Software, Inc., La Jolla, CA). A total of 255 patients (510 joints) were enrolled in the study. Of these, 52 (104 joints) were male and 203 (406 joints) were female, with ages ranging from 18 to 67 (mean age 32.05). Patients with internal derangement of TMJ were included. According to the data obtained from MRI examinations, LPM attachments to the disc condyle complex were categorized into four different types. The most common variation (type II-B) was shown to be two heads with the upper head attached to the disc and condyle, and the lower to the condyle. There was a statistical correlation between the type of LPM attachment and the pathological changes within the joint regarding disc displacement, osteoarthritis, joint effusion, disc degeneration, and condylar translation (*P* = 0.0003, *r* = -0.87, *P* < 0.0001& *r* = 0.29, *P* = 0.0002 & *r* = -0.93, *P* = 0.0061 & *r* = -0.98, and *P* = 0.0004, *r* = -0.54 respectively). The current study shows a statistically significant direct correlation between LPM attachment and TMJ osteoarthritis, while the disc-condyle relationship, joint effusion, disc degeneration, and condylar translation shown significant inverse correlations with LPM attachment patterns.

## Introduction

In recent decades, the lateral pterygoid muscle (LPM) has gained increased attention in research related to TMJ disorders, particularly in comparison to other masticatory muscles. As highlighted by Sicher^1^, the LPM consists of two bellies. The smaller superior head of the LPM (SHLP) extends posteriorly from the infratemporal surface of the greater sphenoid wing in an inferior and lateral trajectory toward the crest of the eminence, where its fibers become more horizontal and attach to the condyle and disc. The larger inferior head of the LPM (IHLP) extends posteriorly and laterally from the outer surface of the lateral pterygoid plate to connect with the pterygoid fovea. Anteriorly, the two bellies are separated by a variably wide gap and converge in front of the TMJ.

The function of the lateral pterygoid muscle (LPM), particularly the superior head of the lateral pterygoid (SHLP), which is integrated into the disc-condyle complex (DCC), is crucial in the biomechanics of the temporomandibular joint (TMJ) disc-condyle complex. Several theories regarding the pathophysiology of internal derangement of the TMJ are predicated on the abnormal functions of the SHLP^2–4^. Anatomical investigations assessing the LPM in cases of internal derangement have corroborated this perspective, noting that the SHLP attaches to both the capsule and the disc^5,6^. Nevertheless, subsequent research indicated that the SHLP is connected to the condyle, capsule, and disc^7,8^. Additional studies have suggested that while the LPM may be securely anchored, it may not insert into the disc^9,10^. The findings from these studies indicate that, anatomically, it is implausible for the SHLP to independently exert a forward pull on the disc, separate from the condyle, which would result in anterior displacement of the disc. Moreover, other studies have indicated that not only the SHLP but also the inferior head of the lateral pterygoid (IHLP) may possess fibres that insert into the disc^11–13^. The variability in the attachment points of the LPM heads has sparked ongoing debate regarding their involvement in the internal derangement of the TMJ.

The proportion of fibers integrated into the disc from the SHLP varies across studies. Naohara^14^ reports that the percentages for the total muscle and the superior head are 3% and 30%, respectively. In contrast, Bittar et al.^12^ indicate that the percentage ranges from 2.6% to 6% of the total muscle, with 31% of the superior head. This finding aligns with the observations made by Naidoo and Juniper^15^, who noted that 29.5% of the SHLP fibres are incorporated into the disc. Furthermore, Zhang et al.^16^ state that merely 10% of these fibres extend to the disc, while 24% are directed toward the joint capsule.

The typical function of the SHLP is linked to the forward movement of the disc alongside the condyle during translation, serving as part of the disc-condyle complex. Conversely, heightened muscle activity of the SHLP has been correlated with the anterior dislocation of the disc^17–19^.

Despite the recognized link between disc displacements and the direct connection of the LPM to the disc, along with the significant correlation between internal derangement and the functions of the LPM, as evidenced by electromyographic studies, the exact function of the LPM in the dynamics of TMJ clicking remains unclear^20,21^. Additionally, the electromyographic evaluation of the SHLP is characterized by a lack of stability and reproducibility; this inconsistency primarily stems from the difficulties in accurately positioning the electrodes within the target muscle belly, especially for the SHLP, due to the LPM’s deep location and diminutive size. If the electrode is inserted too high, too deep, or at an incorrect angle, it may unintentionally be placed within the deep fibers of the anterior temporal muscles or the IHLP^22,23^.

Magnetic resonance imaging (MRI) is currently considered the gold standard for visualizing the temporomandibular joint (TMJ). MRI provides valuable information regarding the position and structure of the disc, offering exceptional soft-tissue resolution while eliminating radiation exposure to the patient. The lateral pterygoid muscle (LPM) can be observed in both coronal and sagittal MR images of the TMJ. Among the different imaging techniques, sagittal and oblique sagittal images of TMJ are essential for the diagnosis of temporomandibular disorders (TMD), as they can reveal the disc, condyle, fossa, and LPM in a single image^24^.

Given the absence of agreement in existing literature and the significant role that evaluation plays in the insertion percentage of the upper head of the lateral pterygoid muscle on the TMJ disc, as well as its implications as a risk factor for anterior disc displacement and the progression of joint pathology, this study aimed to examine the prevalence of the various numbers of lateral pterygoid muscle heads and their types of attachment to the disc-condyle complex. Furthermore, it sought to explore whether these types of attachment are associated with pathological changes in the TMJ.

## Patients and methods

### Study design and population

This observational study analyzes MRI records of the number and pattern of attachments of the lateral pterygoid muscle heads and their association with pathologic changes in the TMJ. We followed the STROBE checklist for reporting our observational study. This study was conducted at the Faculty of Dental Medicine, Al-Azhar University, and the Hegab Academy for Maxillofacial and TMJ Disorders from January 2022 to January 2025.

The patients were screened with an inclusion criterion of TMD, which required an MRI examination to assess internal derangement. The study research protocol was approved by the Hospital IRB, and all participants signed an informed consent agreement. Patients with either TMJ clicking, TMJ locking, restricted jaw movement, or pain in the TMJ region were included in the study. Patients with rheumatoid arthritis, condylar hyperplasia, and congenital craniofacial syndrome, and those who had undergone previous TMJ surgery were excluded from this study.

### MRI evaluation protocol

In the current study, we followed the MRI scanning protocol proposed by Hegab et al.^25^. The MR examinations were performed using a 1.5T unit (Magnetom Vision; Siemens, Erlangen, Germany) with a TMJ surface coil. Six to eight image slices were obtained for each unilateral joint, and a multi-slice examination was carried out for each case. The patients were landmarked using the laser lights, which coincided with the center of the coils. During the MRI examination of both TMJs, the head was positioned with the Frankfort’s line vertical to the table of the MRI device, thus acquiring oblique sagittal plane images vertical to each condylar angulation from the intercuspal position and the maximum opening position. The patients were instructed to keep their heads still and in one position during the examination. All patients underwent a bilateral MRI examination of the TMJ^25^. MRI scans were reviewed by two radiologists with over 20 years of experience in MRI of TMJ disorders. The MRI images were evaluated for the presence or absence of disc displacement. The normal disc position in the sagittal oblique plane was defined as the posterior band of the disc being at the 12 o’clock position relative to the mandibular condyle in the closed mouth position, while in the open mouth position, the thin intermediate band was placed between the mandibular condyle and the articular eminence. Disc displacement with reduction (DDR) was defined as follows: in the closed mouth position, the posterior band of the disc lies anterior to the condyle. In the open-mouth position, the disc returns to the normal position between the condyle and articular eminence. Disc displacement without reduction (DDNR): In the closed mouth position, the posterior band of the disc is anterior to the condyle, while in the open mouth position, the posterior band of the disc stays anterior to the condyle and cannot return to its normal position between the condyle and articular eminence^25^.

### Evaluation of the LPM attachments to DCC

Variation of the number of heads and the attachment pattern of the LPM to DCC was evaluated using MRI in the oblique sagittal and coronal images. The variation in LPM heads and attachment patterns was correlated with pathologic changes in the TMJ.

### TMJ osteoarthritis

TMJ osteoarthritis was diagnosed when one or more of the following changes were noted: flattening of the articular surface, subcortical cyst, surface erosion, osteophytes, flattening of the articular eminence^25^.

### Joint effusion

Joint effusion was assessed on T2WI, manifesting as an area of hyperintensity, which was divided into 3 grades^26^.

Grade 0: No effusion (no bright signal in either joint space).

Grade I: Slight effusion (a bright signal in either joint space that confirmingto the contours of the disc, fossa/articular eminence, and/or condyle).

Grade II: Frank effusion (a bright signal conforming to the disc, fossa/articular eminence, and/or condyle, with a convex configuration in the anterior or posterior recesses).

### Translation of the condyle^26^

Condyle translation was assessed in sagittal oblique images in the open mouth position as follows: 

Hypo: The apex of the condyle translates to less than the apex of the articular eminence.

Normal: The apex of the condyle translates to the apex of the articular eminence.

Hyper: The apex of the condyle translates beyond the apex of the articular eminence^26^.

The pathologic changes of the TMJ were classified according to Hegab Stages^25^ as follows:

Stage 0: Normal MRI study.

Stage 1 A: MRI shows a normal condyle-disc-fossa relationship associated with pathologic changes of the LPM + /joint effusion. Stage 1B: MRI shows a normal condyle-disc-fossa relationship associated with pathologic changes of the disc + /Bone degenerative process of the condyle.

Stage 2 A: MRI shows anterior disc displacement in the closed mouth position with reduction to the normal position in the open mouth position associated with pathologic changes of the LPM + /joint effusion.

Stage 2B: MRI shows anterior disc displacement in the closed mouth position with reduction to the normal position in the open mouth position associated with pathologic changes of the disc + /Bone degenerative process of the condyle.

Stage 2 C: MRI shows anterior disc displacement in the closed mouth position with reduction to the normal position in the open mouth position with condylar hypertranslation.

Stage 3 A: MRI shows anterior disc displacement in the closed mouth position without reduction to the normal position in the open mouth position associated with pathologic changes of the LPM + /joint effusion.

Stage 3B: MRI shows anterior disc displacement in the closed mouth position without reduction to the normal position in the open mouth position, associated with pathologic changes of the disc + /Bone degenerative process of the condyle.

Stage 3 C: MRI shows anterior disc displacement in the closed mouth position without reduction to the normal position in the open mouth position, associated with normal translation movement of the mandibular condyle (no limitation of the mouth opening).

Stage 4: MRI shows posterior disc displacement.

### Statistical analysis

A post hoc power analysis was designed to determine the study’s power. The MedCalc version 12.3.0.0 program was used for sample size calculations, based on a 95% confidence interval and a study power of 80% with an α of 5%. According to a previous study (Fujita et al. 2001)^13^ The power was found to be 0.97 (97%), indicating that the sample size was adequate. The sample size calculation was performed using G*Power version 3.1.9.2. The significance level was set at 0.05. The numerical data were explored for normality by examining the distributions of the data, calculating the means and medians, and using tests of normality (e.g., the Kolmogorov Smirnov and Shapiro–Wilk tests). The age data, presented as the mean and standard deviation (SD) values. Co-variance effects such as gender, age, type of insertion of the muscle upper head, articular disc morphology, presence/absence of effusion, and position of the articular disc (normal/anterior dislocation with or without reduction) were analyzed by linear regression (α = 5%). The data were analyzed using Instat statistical software (GraphPad Software, Inc., La Jolla, CA)^25^.

## Results

A total of 255 patients (510 joints) were enrolled in the study. Of these, 52 patients (104 joints) were male and 203 (406 joints) were female, with ages ranging from 18 to 67 years (mean age: 32.05 years). Those with internal derangement of the TMJ were enrolled in the study.

The insertion type of the heads of the lateral pterygoid muscle was evaluated in the oblique sagittal MRI. The Types of lateral pterygoid muscle attachments to the DDC were categorized into four different types according to the MRI evaluation:

Type I: A single head attached to the DCC.

Type II: Two heads, further subdivided into.

Type II-A: Upper head attached to the disc; lower head to the condyle.

Type II-B: Upper head attached to both the disc and condyle; lower head to the condyle.

Type III: Three heads, subdivided into.

Type III-A: Upper head attached to the disc; lower two to the condyle.

Type III-B: Upper head attached to both the disc and condyle, lower two to the condyle.

And Type IV: Four heads, with the upper head attached to the disc and the lower three to the condyle.

**Fig. 1:**
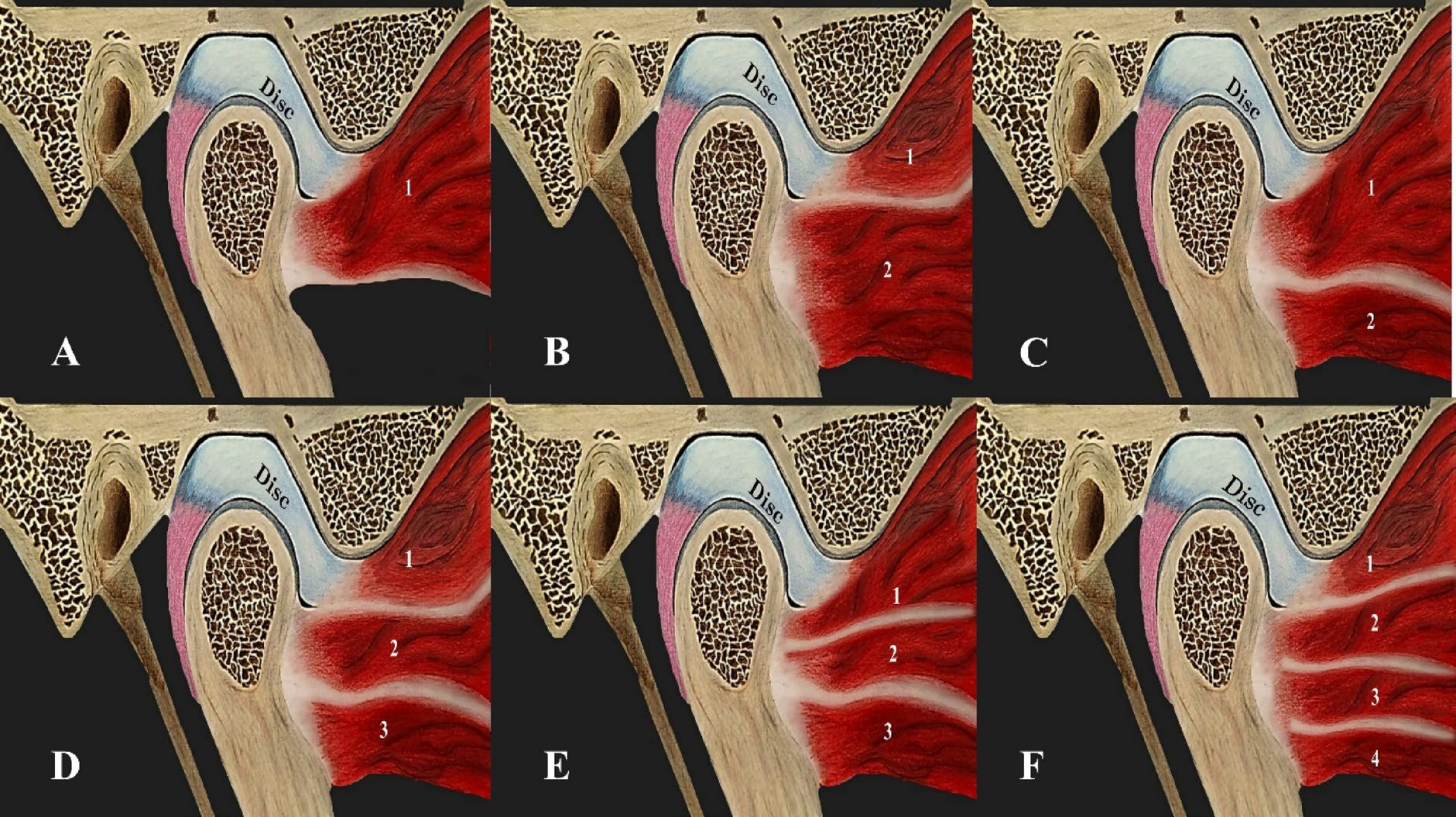
An Illustration representing the number of LPM heads and the different patten of attachment to the disc-condyle complex. (**A**) represent Type I with one head of lateral pterygoid muscle head attached to the DCC. (**B**) represent Type II-A with two heads of lateral pterygoid muscle, the upper attached to the disc and the lower to the condyle. (**C**) represent Type II-B with two heads of lateral pterygoid muscle, with upper attached to the disc and condyle and the lower to the condyle. (**D**) represent Type III-A with three heads of lateral pterygoid muscle, the upper attached to the disc and the lower two attached to the condyle. (**E**) represent Type III-B with three heads of lateral pterygoid muscle, the upper attached to the disc and condyle and the lower two attached to the condyle. (**F**) represent Type IV with four heads of lateral pterygoid muscle, the upper attached to the disc and the lower three attached to the condyle.

Table 1; Fig. 1 represent an illustration of the number of LPM heads and the pattern of attachment to the disc-condyle complex.

**Fig. 2:**
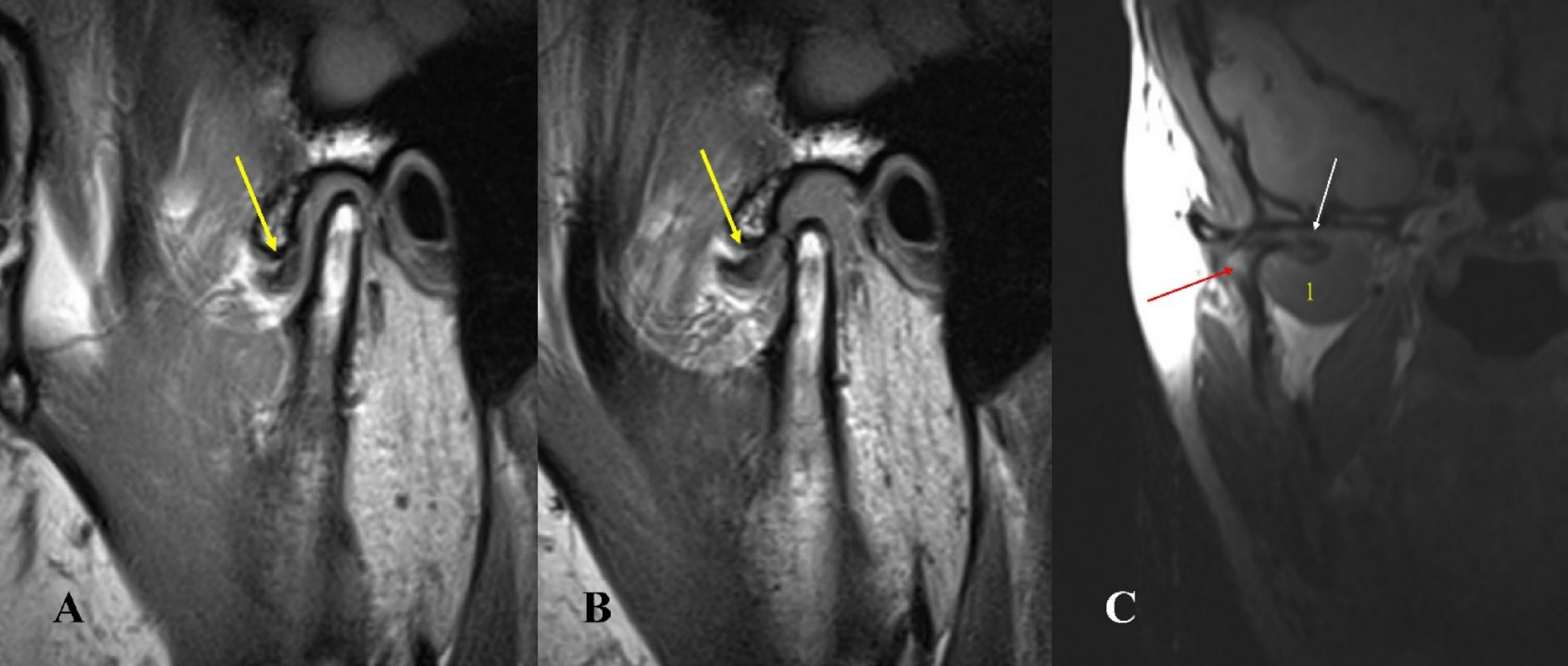
Oblique sagittal T1-weighted images represent Type I with one head of lateral pterygoid muscle head attached to the DCC associated with disc displacement without reduction and severe form of osteoarthritis.

**Fig. 3:**
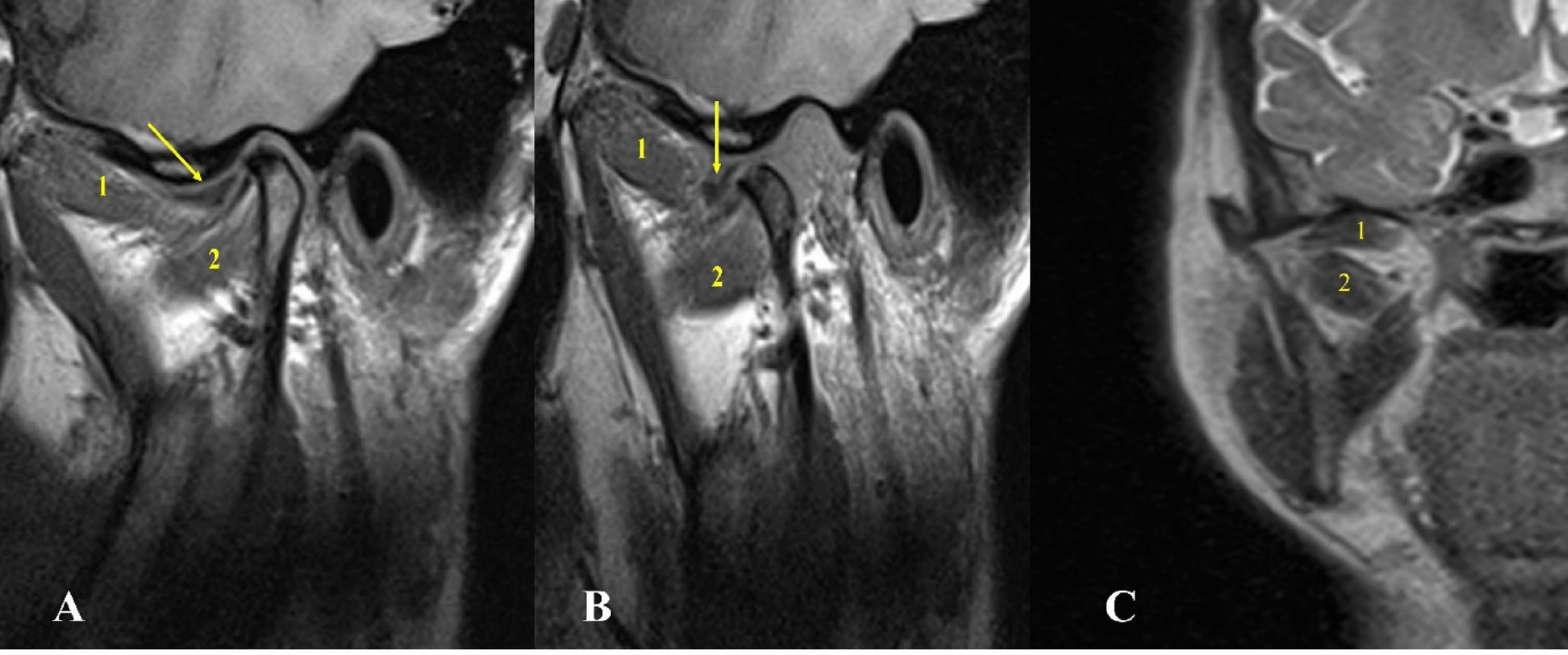
Oblique sagittal T1-weighted images represent Type II-A with two heads of lateral pterygoid muscle, the upper attached to the disc and the lower to the condyle associated with disc displacement without reduction and condylar erosion, osteophyte and bone marrow oedema.

**Fig. 4:**
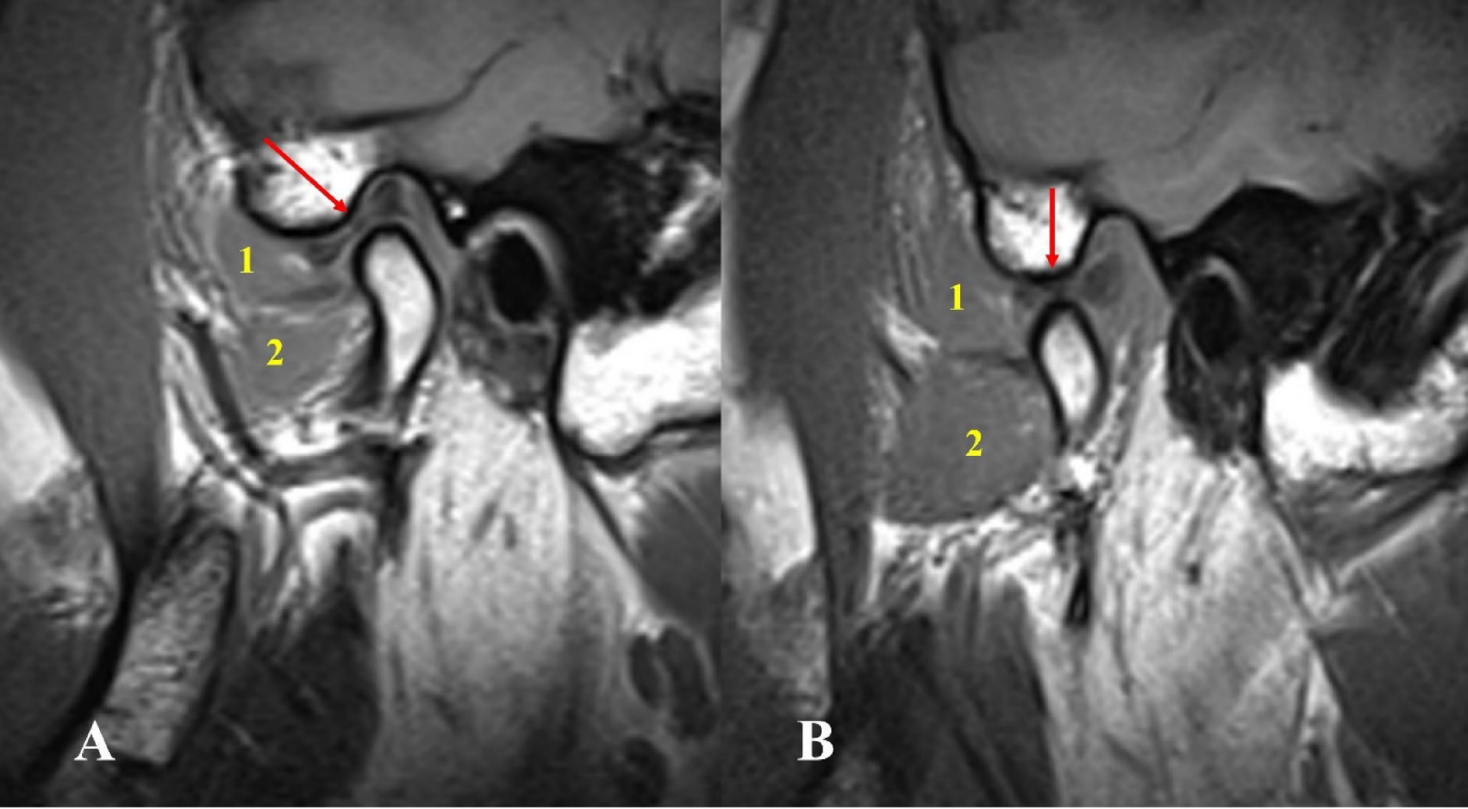
Oblique sagittal T1-weighted images represent Type II-B with two heads of lateral pterygoid muscle, with upper attached to the disc and condyle and the lower to the condyle.

**Fig. 5:**
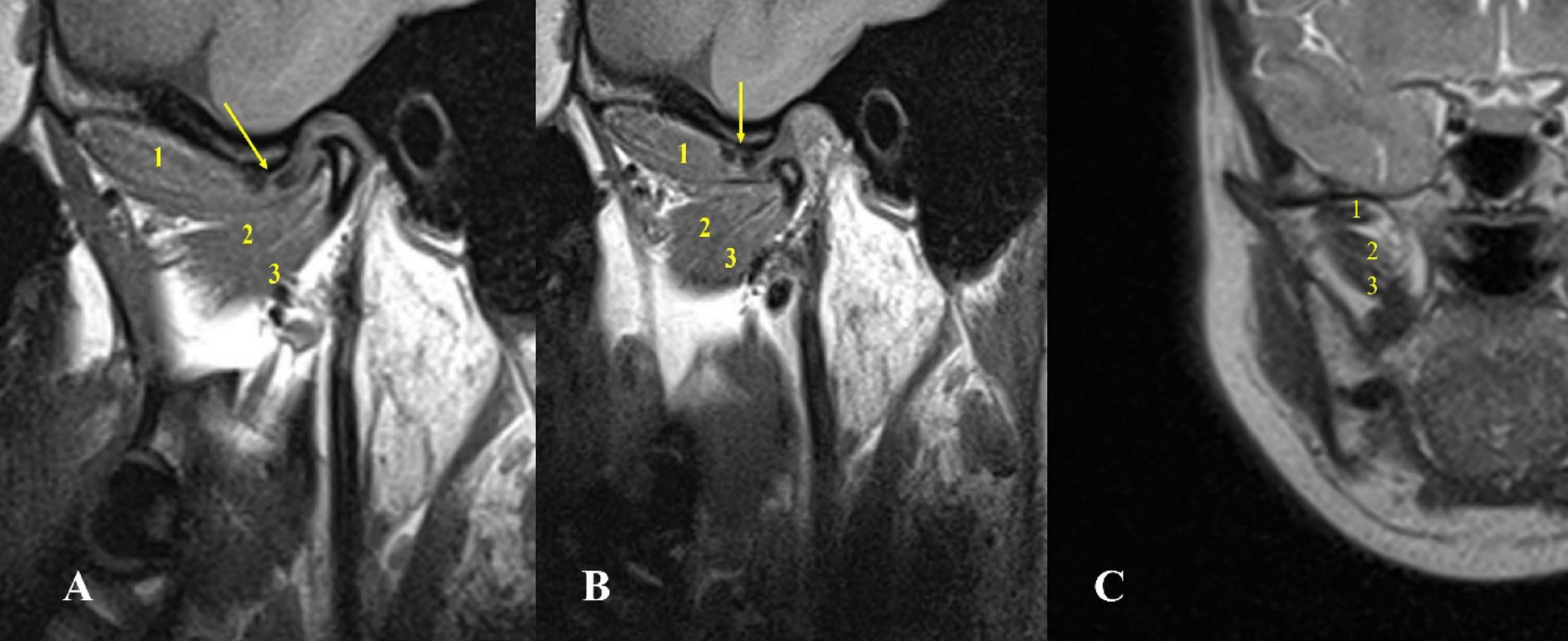
Oblique sagittal T1-weighted images represent Type III-A with three heads of lateral pterygoid muscle, the upper attached to the disc and the lower two attached to the condyle associated with disc displacement without reduction and condylar erosion, osteophyte and condylar resorption.

**Fig. 6:**
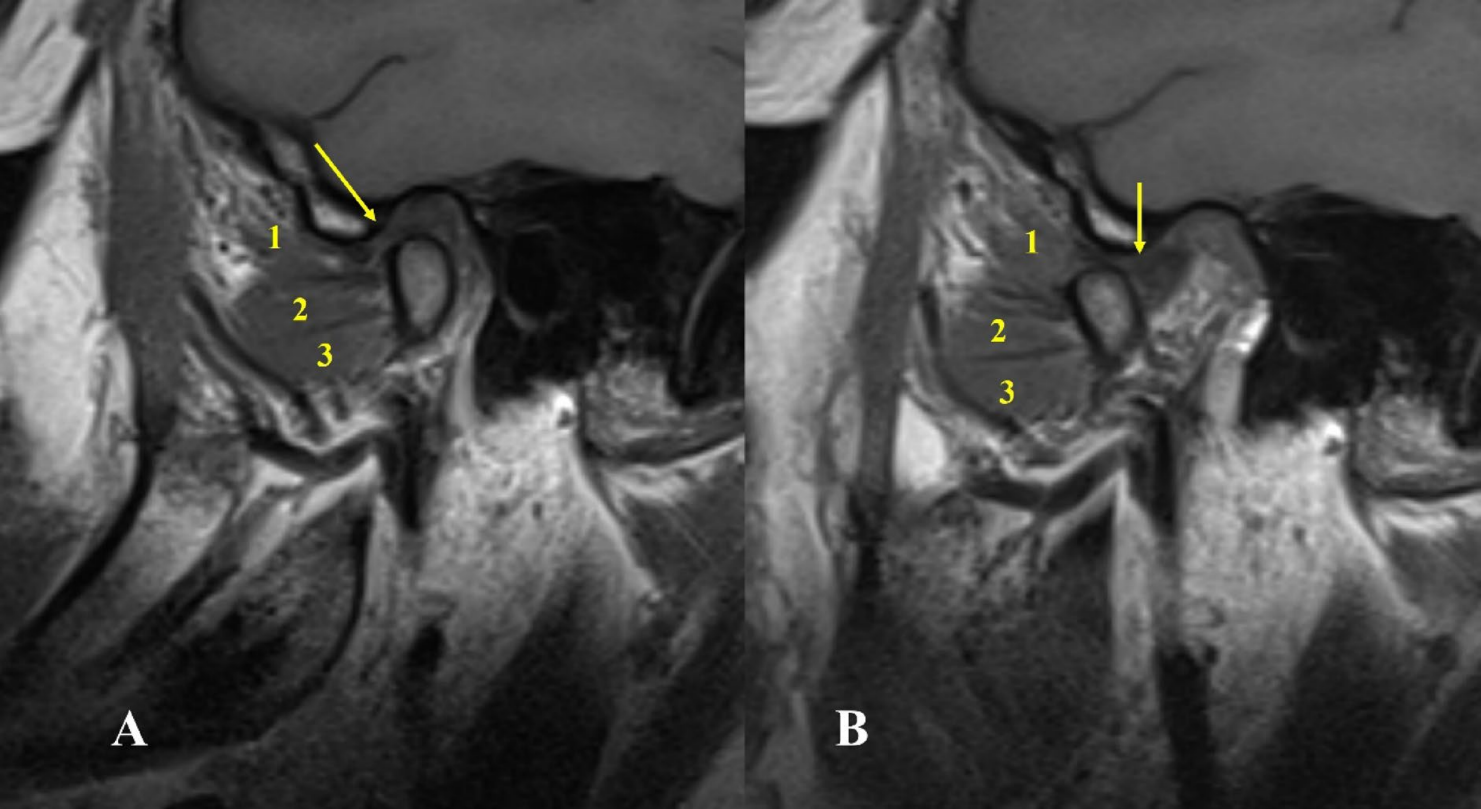
Oblique sagittal T1-weighted images represent Type III-B with three heads of lateral pterygoid muscle, the upper attached to the disc and condyle and the lower two attached to the condyle.

**Fig. 7:**
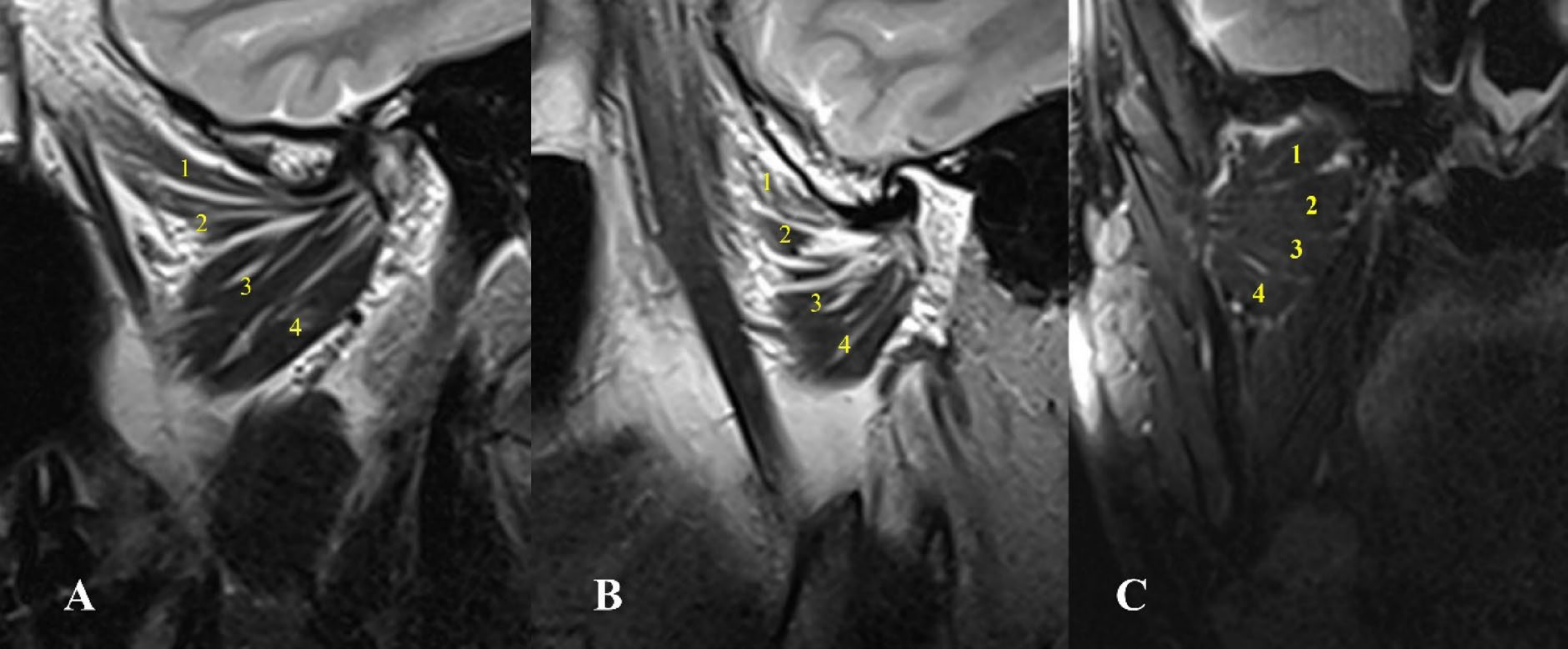
Oblique sagittal T1-weighted images represent Type IV with four heads of lateral pterygoid muscle, the upper attached to the disc and the lower three attached to the condyle associated with condylar erosion, and condylar resorption.

The prevalence of different types of lateral pterygoid muscle attachments to the disc condyle complex was as follows: Type I: 6 joints (1.17%), Type II-A: 30 joints (5.8%), Type II-B: 339 joints (66.47%), Type III-A: 10 joints (1.96%), Type III-B: 122 joints (24%) and Type IV: 3 joints (0.6%). The most common variation (type II-B) was characterized by two heads, with the upper head attached to the disc and condyle, and the lower head attached to the condyle. followed by Type III-B with three heads and the upper head attached to the disc and condyle, and the lower two attached to the condyle. While the lowest variation was Type IV (0.6). Type I was shown to be associated with aggressive pathologic changes within the TMJ in all the cases included: DDNR, osteoarthritis, joint effusion, and condylar hypomobility. (Table 2; Figs. 2, 3, 4, 5, 6 and 7 represent Oblique sagittal T1-weighted images for different types of LPM head numbers and attachment patterns).

**Table 1:** Represents the number of LPM heads and the pattern of attachment to the disc-condyle complex.

Type	Subtype	Type of attachment
Type I (one head)	–	One head attached to the DCC
Type II (two heads)	Type IIA	The upper attached to the disc and the lower to the condyle.
	Type IIB	The upper attached to the disk and condyle and the lower to the condyle.
Type III (three heads)	Type IIIA	The upper attached to the disc and the lower two attached to the condyle.
	Type IIIB	The upper attached to the disc and condyle and the lower two attached to the condyle.
Type IV (four heads)	Type IV	The upper attached to the disc and the lower three attached to the condyle.

A statistically significant direct correlation was found between the number and pattern of LPM attachments and osteoarthritis of the TMJ (*P* < 0.0001; *r* = 0.29; *P* = 0.0002). In contrast, significant inverse correlations were found between LPM attachment patterns and the disc-condyle relationship (*P* = 0.0003; *r* = − 0.87), joint effusion (*P* = 0.0002; *r* = − 0.93), disc degeneration (*P* = 0.0061; *r* = − 0.98), and condylar translation (*P* = 0.0004; *r* = − 0.54) (Table 2). Regarding pathologic changes in the TMJ, 19 joints were classified as Hegab Stage 0, 61 joints as Hegab Stage 1 A, 57 joints as Hegab Stage 1B, 46 joints as Hegab Stage 2 A, 134 joints as Hegab Stage 1B, 47 joints as Hegab Stage 2 C, 9 joints as Hegab Stage 3 A, 124 joints as Hegab Stage 3B, and 13 joints as Hegab Stage 3 C. No cases of posterior disc displacement (Hegab Stage 4) were observed. Table 3.

**Table 2:** Prevalence of joint pathologic changes with different types of LPM attachment.

Study group		Type I	Type II-A	Type II-B	Type III-A	Type III-B	Type IV	*P*-value
Sample size		6 (1.17%)	30 (5.8%)	339 (66.47%)	10 (1.96%)	122 (24%)	3 (0.6%)	
Disc-condyle relationship	Normal	-	4	88	-	43	-	0.0003***
	DDR	-	14	167	7	47	-	
	DDNR	6	12	84	3	32	3	
Osteoarthritis	Normal	-	6	139	5	48	-	< 0.0001***
	Flattening	-	4	32	-	7	-	
	Erosion	6	10	101	2	39	3	
	Osteophyte	4	21	50	3	34	-	
	Sub-condylar cyst	4	6	17	8	4	-	
	Surface irregularity	6	15	185	9	53	3	
	Articular eminence Flattening	5	18	132	7	31	3	
	Combination	6	8	74	8	37	3	
Joint effusion	Grade 0	-	16	270	8	96	2	0.0002***
	Grade I	2	6	56	2	20	1	
	Grade II	4	8	13	-	6	-	
Disk degeneration	Present	6	25	206	4	66	3	0.0061**
	Absent	-	5	133	6	56	-	
Condylar translation	Normal	-	6	121	1	48	-	0.0004***
	Hypo	6	18	171	6	62	3	
	Hyper	-	6	47	3	12	-	

**Table 3:** Demographic features and Hegab stage in each type of LPM attachment.

Study group		Type I	Type II-A	Type II-B	Type III-A	Type III-B	Type IV
Sample size		6 (1.17%)	30 (5.8%)	339 (66.47%)	10 (1.96%)	122 (24%)	3 (0.6%)
Gender	Male	1	-	61	4	38	-
	Female	5	30	278	6	84	3
Mean age		40.83 ± 12.1	32.87 ± 7.1	31.65 ± 10.12	31.50 ± 6.8	31.94 ± 7.8	39.67 ± 8.9
HEGAB stage	Stage 0	-	-	12	-	7	-
	Stage 1 A	-	-	51	-	10	-
	Stage 1B	-	5	25	-	27	-
	Stage 2 A	-	-	38	-	8	-
	Stage 2B	-	5	94	-	35	-
	Stage 2 C	-	6	35	3	3	-
	Stage 3 A	-	-	9	-	-	-
	Stage 3B	6	14	62	7	32	3
	Stage 3 C	-	-	13	-	-	-

## Discussion

The lateral pterygoid muscle is crucial for mandibular movement and has been examined from various perspectives, including biology and functional anatomy. Numerous studies have been conducted regarding the attachment of the lateral pterygoid muscle fibers to the temporomandibular joint, particularly at the insertion point, yet the findings have not yielded consistent results. In discussing the lateral pterygoid muscle’s involvement in temporomandibular joint disorders, it is essential to detail the number of lateral pterygoid muscles and their attachment patterns to the articular disc^27–29^.

Magnetic resonance imaging (MRI) is presently considered the gold standard for imaging temporomandibular joints (TMJ). MRI provides insights into the disc’s position and morphology through its high soft-tissue resolution, all while minimizing radiation exposure for the patient. The lateral pterygoid muscle (LPM) is recognized for its anatomical variability, especially regarding the number of heads it possesses. Traditional anatomical texts typically describe the LPM as consisting of two heads: a superior head (sphenoid, SLPM) and an inferior head (pterygoid, ILPM). There has been considerable speculation regarding the function of the LPM in the context of TMJ internal derangement.

The hypothesis regarding the function of the LPM encompasses factors such as muscle hyperactivity, muscle hypoactivity, inadequate coordination between the actions of the two heads, and the insertion patterns of the heads of the LMP into the DDC^9,11,17,18^. The anatomical literature generally concurs that the LMP consists of two heads; however, numerous authors have documented the existence of a third head^13,15,18,19^.

The belief that hyperactivity of the superior head of the lateral pterygoid muscle causes degenerative arthritis changes in the temporomandibular joint is not evidence-based. However, in the current study, all cases with a single head of the LPM were linked to disc displacement without reduction and a severe form of osteoarthritis (Hegab stage 3B). Additionally, Type II-A and Type III-A. cases, where SHLP inserted directly into the disc, were also associated with significant joint pathology.

Regarding the attachment pattern of the LPM heads to the DCC, considerable attention has been directed towards the superior head of the LPM in numerous reports that seek to elucidate the issues related to the TMJ and the anterior displacement of the joint disc. The attachment of the superior head of the LPM, whether to the disc or the condyle, has been a topic of discussion among researchers for many years^30–35^.

Most studies and textbooks suggest that the LPM is a two-headed muscle, while some recent research has identified a third head of the LPM, with the majority of the superior head inserting into the condyle and a small portion often attached to the anteromedial aspect of the capsule and disc^6,13,19^. However, only a limited number of articles have indicated that it is a one-headed muscle^14^.

In the present study, we report four different types of LPM heads: one with a single head and another variation with four heads. The observed association between the single-headed LPM and severe joint pathology raises an important question: does this reflect a consequence of joint disease leading to muscle degeneration, or is the single-headed configuration itself a high-risk anatomical variant? we believed from our results that the single-headed configuration itself a high-risk anatomical variant because we find in our study muscle degeneration that has no effect on the number of the LPM heads. The causal relationship warrants further investigation.

The present study confirms that the LPM exhibits variable attachment patterns to the disc and DCC, which appear to play an important role in TMJ internal derangement.

The anteromedial attachment of the LPM to the DCC can explain why most of the disc displacements were in the anteromedial direction, and it supports the role of the LPM in the displacement of the TMJ articular disc.

## Conclusion

According to the results of the current study, LPM attachments to the disc condyle complex were categorized into four different types. The most common variation (type II-B) was shown to be two heads with the upper head attached to the disc and condyle, and the lower to the condyle. Based on the record of the current study, there was a statistically significant direct correlation between the number and pattern of LPM attachment and TMJ osteoarthritis. Meanwhile, the disc-condyle relationship, joint effusion, disk degeneration, and condylar translation had a statistically significant inverse correlation with the number and pattern of LPM attachment.

## Data Availability

The datasets used and analysed during the current study are available from the corresponding author on reasonable request.
